# Yersinopine is a primary zinc acquisition system for *Yersinia pseudotuberculosis*

**DOI:** 10.1128/mbio.02000-26

**Published:** 2026-09-17

**Authors:** Mahendar Kadari, Casey Weber, Thomas E. Kehl-Fie, Robert D. Perry, Juan C. Ortiz-Marquez, Oleg V. Tsodikov, Sylvie Garneau-Tsodikova, Matthew B. Lawrenz

**Affiliations:** 1 Department of Microbiology and Immunology, University of Louisville School of Medicine 12254, Louisville, Kentucky, USA; 2 Department of Microbiology and Immunology, University of Iowa Carver College of Medicine 4083, Iowa City, Iowa, USA; 3 Department of Microbiology, Immunology, and Molecular Genetics, College of Medicine, University of Kentucky, Lexington, Kentucky, USA; 4 Division of Infectious Diseases, Boston Children's Hospital, Harvard Medical School 1811 , Boston, Massachusetts, USA; 5 Department of Pharmaceutical Sciences, College of Pharmacy, University of Kentucky 4530, Lexington, Kentucky, USA; 6 Center for Predictive Medicine for Biodefense and Emerging Infectious Diseases, University of Louisville 127379, Louisville, Kentucky, USA; NYU Langone Health, New York, New York, USA

**Keywords:** *Yersinia*, nutritional immunity, metalloproteins, zinc, metal homeostasis

## Abstract

Y*ersinia pestis* is a vector-borne pathogen that evolved from the enteric pathogen *Yersinia pseudotuberculosis*. Despite different routes of infection, both pathogens need to overcome host-mediated zinc limitation. *Y. pestis* relies on the transporter ZnuABC and the metallophore yersiniabactin, but surprisingly, disruption of these systems in *Y. pseudotuberculosis* did not limit growth during zinc limitation, suggesting the presence of additional mechanisms. Here, we identify the opine-type metallophore yersinopine, encoded by the *cnt* operon, as a primary zinc uptake system in *Y. pseudotuberculosis*. While a *znuA ybtX* mutant was not attenuated for growth in metal-limited medium, *cnt znuA* and *cnt ybtX* mutants both had significant growth defects. Moreover, a *znuA ybtX cnt* mutant was completely attenuated for growth, which was rescued by zinc but not iron supplementation. While the *cnt* operon is present in *Y. pestis*, growth of analogous *cnt* mutants was not altered. Comparison of the *cnt* operons indicated the presence of two frameshift mutations in the predicted yersinopine importer *cntQ* in *Y. pestis*, suggesting that it is a pseudogene. Indeed, introduction of the same mutations into *cntQ* in *Y. pseudotuberculosis znuA ybtX* phenocopied a *znuA ybtX cnt* mutant. Finally, yersinopine also contributed to *Y. pseudotuberculosis* fitness in the presence of calprotectin and during infection in the murine model of yersiniosis. These data establish yersinopine as a primary zinc acquisition system in *Y. pseudotuberculosis* that is not required for *Y. pestis* to overcome zinc-mediated nutritional immunity and show divergent evolution in zinc acquisition strategies in these closely related species.

**IMPORTANCE:**

Zinc sequestration by the host is a major component of nutritional immunity that restricts bacterial growth during infection. Here, we identify the opine metallophore yersinopine as a primary zinc acquisition system in *Yersinia pseudotuberculosis*. Yersinopine promotes fitness during calprotectin-mediated zinc starvation and contributes to virulence during infection. In contrast, yersinopine does not support zinc uptake in *Yersinia pestis* due to inactivating mutations in the protein required for uptake of zinc-bound yersinopine. These findings establish yersinopine-dependent zinc acquisition as a critical virulence factor in *Y. pseudotuberculosis* and reveal a previously unrecognized evolutionary divergence in metal acquisition strategies among closely related *Yersinia* species.

## INTRODUCTION

Transition metals, such as iron (Fe), zinc (Zn), manganese (Mn), and copper (Cu), serve as cofactors for numerous proteins and enzymes that are essential for many biological processes (1–3). In bacteria, these metals are critical for growth, transcriptional regulation, and the expression of virulence factors. However, both metal scarcity and excess can impair bacterial physiology, making the maintenance of metal homeostasis a tightly regulated process in bacteria (1, 4). At the host-pathogen interface, competition for metal availability has driven the evolution of sophisticated acquisition, sequestration, and detoxification strategies on both sides, shaping microbial fitness and host resistance to infection (1, 4–6). A central host defense strategy, termed nutritional immunity, restricts pathogen access to essential transition metals through sequestration by high-affinity metal-binding proteins such as transferrin, lactoferrin, and calprotectin (1, 4–7). During inflammation, calprotectin (CP) is abundantly released by infiltrating neutrophils (8). CP is a heterodimer composed of S100A8 and S100A9 and constitutes approximately 40%–50% of the protein content of the neutrophil cytoplasm (8–11). As a dimer, CP has two metal-binding sites, a conserved 3HisAsp site that binds Zn and a 6His site that binds Zn, Mn, and other metals (12). Several studies have shown that CP serves as a dominant mechanism for Zn and Mn sequestration during infection (8, 9, 11), including in the inflamed gut, where it contributes to a profoundly metal-limited niche (13, 14).

**Fig 1:**
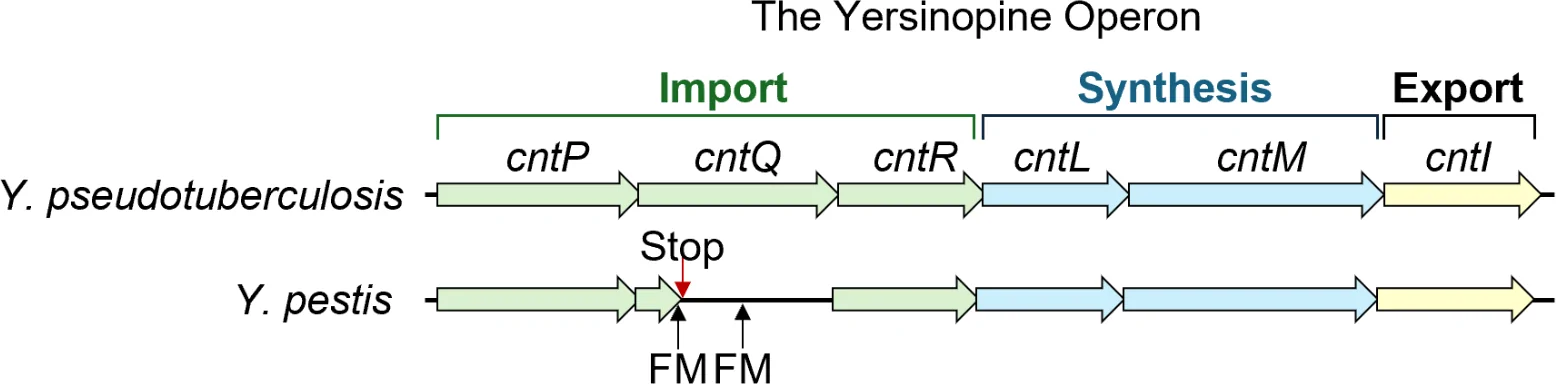
Comparison of genetic organization of the *cnt* operon between *Y. pseudotuberculosis* and *Y. pestis*. Predicted gene functions are indicated. Frameshift mutations (FM) resulting from deletions of T217 and T566 are denoted with arrows, and the gene truncation resulting from the premature stop codon at T217 (Stop) in *Y. pestis cntQ* is highlighted.

To counter nutritional immunity, bacteria have evolved a diverse array of metal acquisition systems, including ATP-binding cassette (ABC) transporters, Zrt- and Irt-like protein (ZIP) family metal ion transporters, and secreted metallophores. In the case of Zn acquisition, ZnuABC and the related AdcABC system are highly conserved ABC importers essential for overcoming Zn limitation during infection by *Salmonella enterica*, *Campylobacter jejuni*, *Haemophilus ducreyi*, *Moraxella catarrhalis*, and uropathogenic *Escherichia coli* (9, 15–19). In addition, ZupT, a member of the ZIP family of proteins, has also been implicated in Zn uptake in *E. coli* and *S. enterica* (20, 21). Recently, the siderophore yersiniabactin (Ybt), which is essential for Fe acquisition in the *Yersiniae* (22), has been shown to also contribute to Zn acquisition in both *Yersinia pestis* and *E. coli* Nissle (23, 24). Several bacteria also produce nicotianamine-like metallophores, structurally analogous to plant and fungal nicotianamine, that facilitate the acquisition of a broad range of transition metals, including Zn (25, 26). These include staphylopine in *Staphylococcus aureus*, pseudopaline in *Pseudomonas aeruginosa*, and yersinopine in *Y. pestis* and *Yersinia pseudotuberculosis* (27, 28). The biosynthesis and transport of these nicotianamine-like metallophores are mediated by a highly conserved *cnt* (cobalt nickel transporter) operon, which encodes proteins required for metallophore synthesis (*cntL* and *cntM*), export (*cntI*), and uptake of the metal-bound metallophore (*cntP*, *cntQ*, and *cntR*) (Fig. 1) (29). Expression of the *cnt* genes is primarily induced by Zn limitation and is tightly regulated by Zur (Zn uptake regulator) in *Pseudomonas* and *Yersinia* (28, 30–33). However, in *S. aureus*, *cnt* gene expression responds to both Zn and Fe availability and is regulated by both Zur and Fur (Fe uptake regulator) (34). Staphylopine and pseudopaline have been directly linked to Zn acquisition (28, 35), but despite being regulated by Zur, a previous study suggested that yersinopine is not required for Zn limitation but instead is involved in Fe acquisition in *Y. pseudotuberculosis* (32). While these different mechanisms can help pathogens compete for Zn with the host, many pathogens have evolved to encode multiple Zn acquisition systems. For example, while *Y. pestis* can use Ybt to acquire Zn during infection, it also encodes a functionally redundant ZnuABC system (23). Similarly, *Klebsiella pneumoniae* encodes for two ABC transporters to acquire Zn (ZnuABC and ZniCBA) (36), and hypervirulent strains also encode Ybt (37). These examples of encoding redundant systems underscore the importance of metal acquisition to bacterial virulence and suggest that the energy cost of multiple systems can significantly improve the fitness of a variety of pathogens during infection.

*Y. pseudotuberculosis* is an enteric pathogen closely related to *Y. pestis*, typically causing a self-limiting gastroenteritis called yersiniosis (38, 39). While *Y. pseudotuberculosis* encounters Zn restriction during infection, it remains unclear how the bacteria overcome Zn-mediated nutritional immunity. Like *Y. pestis*, *Y. pseudotuberculosis* encodes both ZnuABC and Ybt. YezP, a type VI secretion system cluster 4 (T6SS-4) secreted protein, also appears to bind Zn, but there is no evidence that YezP provides a growth advantage in Zn-limited medium (40). Instead, the data suggest that YezP-mediated Zn binding (in the *znuBC* background) helps to protect *Y. pseudotuberculosis* against oxidative stress (40). Therefore, the Zn acquisition systems required to overcome Zn sequestration during infection have yet to be identified in *Y. pseudotuberculosis*. In this study, we demonstrate that ZnuABC and Ybt, which function as redundant Zn acquisition systems in the closely related *Y. pestis*, are dispensable for *Y. pseudotuberculosis* growth under Zn limitation. Instead, we identify the opine metallophore yersinopine as an additional Zn acquisition system in *Y. pseudotuberculosis* that contributes to fitness in the mouse model of infection.

## MATERIALS AND METHODS

### Bacterial strains

All bacterial strains used in this study are listed in Tables S1 and S2. Mutations were generated in the *Y. pseudotuberculosis* IP32953 strain obtained from Dr. Ralph Isberg (41) or the *Y. pestis* KIM6^+^ background [pCD1^−^ pMT^+^ pPCP1^+^ Pgm^+^] (42). *Escherichia coli* S17 was used as a donor strain for genetic manipulation (43). *Y. pestis* was routinely grown in Difco brain heart infusion (BHI) broth (BD Biosciences), while *Y. pseudotuberculosis* and *E. coli* S17 were grown in Miller Luria-Bertani (LB) medium (BD Biosciences). When necessary, the following antibiotics were added to the cultures: kanamycin (25 µg/mL for *Y. pestis* and 50 µg/mL for *Y. pseudotuberculosis* and *E. coli*), irgasan (2 µg/mL), or polymyxin B (25 µg/mL).

### Cloning, site-directed mutagenesis, and generation of mutants

All primers used in this study are listed in Table S3. Mutant strains of *Y. pseudotuberculosis* and *Y. pestis* were generated as previously described (43). Briefly, to generate in-frame deletions, genomic regions flanking the target gene were amplified by PCR and assembled into the pSR47s suicide plasmid in *E. coli* S17 for conjugation (44). Following conjugation with the appropriate *Yersinia* species, candidate recombinants were isolated by antibiotic selection and resolved through sucrose-based counterselection to select for plasmid loss. Resulting colonies were screened for the desired allelic exchange, and successful deletions were verified by PCR. For site-directed mutagenesis, the target genes, together with their flanking regions, were first cloned into pSR47s, and specific nucleotide insertions or deletions were introduced using mutagenic primers. Modified constructs were confirmed by sequencing and integrated into the chromosome using the same allelic-exchange approach described above. All mutants used in this study were verified by whole-genome sequencing (Plasmidsaurus).

### Growth curve kinetics under Zn-depleted and Zn-replete conditions

To avoid 37°C growth restrictions, growth curves were conducted using *Yersinia* strains cured of the virulence plasmid (pYV or pCD1). Growth curves were performed in metal-depleted Chelex-treated M9 medium (Na_2_HPO_4_, 6 g/L; KH_2_PO_4_, 3 g/L; NaCl, 0.5 g/L; and NH_4_Cl, 1 g/L). For *Y. pseudotuberculosis*, the medium was supplemented with 1 mM MgSO_4_ and 1 µM FeSO_4_ for Zn restriction experiments, and with 10 µM ZnCl_2_ or FeSO_4_ for Zn- or Fe-replete conditions, respectively. For *Y. pestis*, the medium was supplemented with 20 mM MgSO_4_, 1 µM FeSO_4_, and 0.6 µM ZnCl_2_ for Zn restriction experiments, and with 10 µM ZnCl_2_ for Zn-replete conditions. Prior to growth in M9-medium, overnight cultures were grown at 26°C in either LB or BHI medium on a roller drum for *Y. pseudotuberculosis* and *Y. pestis*, respectively. Cultures were diluted to an OD_600_ equivalent of 0.1 and passaged twice (for 8 and 15 h) in Chelex-treated M9 medium in metal-free Falcon tubes (Avantor) at 37°C to deplete intracellular Zn stores. Following Zn depletion, bacteria were resuspended in fresh Chelex-treated M9 medium with or without Zn supplementation and adjusted to a final concentration of 1 × 10^7^ CFU/mL. Two hundred microliters of bacterial suspensions was added into each well (*n* = 3) of a U-bottom, crystal clear 96-well plate (Greiner Bio-One). Absorbance at OD_600_ was measured at 37°C every 30 min for 18 h using a BioTek Cytation 1 (Agilent). Growth curves were independently repeated three times.

### Evaluation of *Y. pseudotuberculosis* growth in the presence of calprotectin

Recombinant calprotectin and its variant were overexpressed in *E. coli* and purified as previously described (11, 45, 46). Growth assays for *Y. pseudotuberculosis* in the presence of increasing concentrations of calprotectin were performed as described previously, with minor modifications (47). Briefly, overnight cultures of *Y. pseudotuberculosis* grown in LB at 26°C were diluted 1:100 into Chelex-treated LB and incubated at 37°C for 4 h. A total of 1 × 10^6^ CFU were transferred to individual wells of a U-bottom, crystal clear 96-well plate (Greiner Bio-One) containing 38% LB and 62% calprotectin buffer (20 mM Tris, pH 7.5, 100 mM NaCl, and 3 mM CaCl_2_), supplemented with the indicated concentrations of calprotectin or the metal-binding variant (*n* = 3). Plates were incubated at 37°C with OD_600_ measured every 30 min as described above. Growth curves were independently repeated three times.

### Animal studies

*Y. pseudotuberculosis* was cultured overnight in LB broth at 26°C on a roller drum. Bacteria were washed into phosphate-buffered saline at a concentration of 1  ×  10^9^ CFU/mL. C57BL/6J mice were administered 100 µL of the standardized suspension (1  ×  10^8^ CFU) by oral gavage. Inocula were confirmed by serial dilution and plating on LB agar plates. Following infection, animals were monitored every 12 h for 16 days for the development of moribund disease. Moribund animals that met predefined endpoint criteria were humanely euthanized and scored as succumbing to infection 12 h later. Kaplan-Meier survival curves and log-rank tests were used to determine significant differences in median survival.

### Statistical analysis

*In vitro* assays represent data from three independent biological experiments. Animal experiments were conducted twice. All data are presented as means ± standard deviations. Statistical significance was determined using one-way ANOVA with Dunnett’s test (Zn assays), two-way ANOVA with Tukey’s test (CP assays), and the log-rank (Mantel-Cox) test (survival). All statistics were calculated using GraphPad Prism software. For clarity, only statistically significant group comparisons are denoted within each figure. Any comparison in which the *P* value was >0.05 was not considered statistically significant and thus not denoted.

## RESULTS

### ZnuABC and yersiniabactin are redundant Zn acquisition systems in *Y. pestis* but dispensable for *Y. pseudotuberculosis* growth under Zn restriction

**Fig 2:**
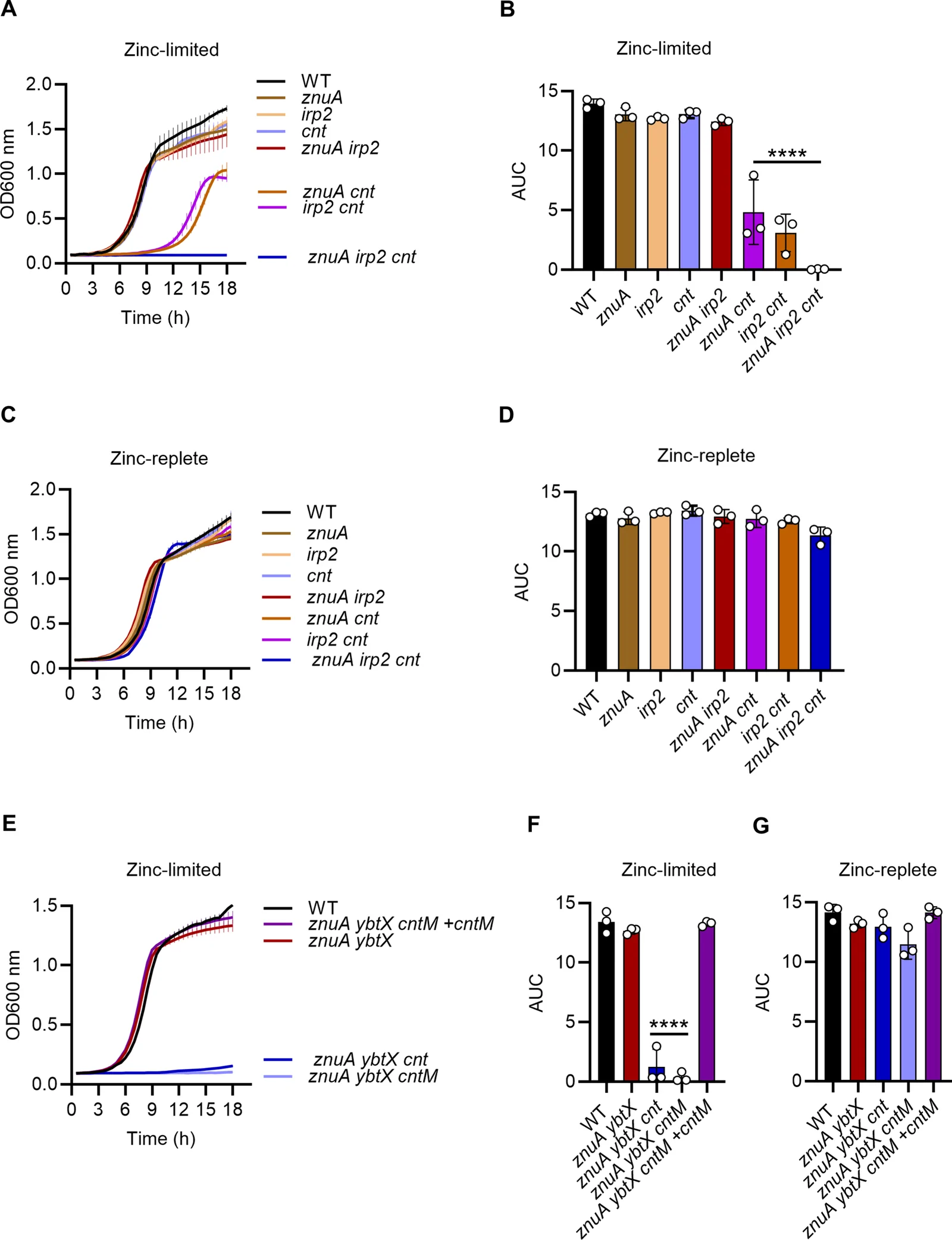
Yersinopine functions as a redundant Zn acquisition system in *Y. pseudotuberculosis*. (**A**) Representative growth curve of *Y. pseudotuberculosis* (WT) and indicated mutant strains under Zn-limited conditions. (**B**) Area under the curve (AUC) from three biologically independent growth curves performed as described for panel **A**. (**C**) Representative growth curve of the indicated strains under Zn-replete conditions (addition of 10 µM ZnCl_2_). (**D**) AUC from three biologically independent growth curves performed as described for panel **C**. (**E**) Representative growth curve of the indicated strains under Zn-limited conditions. (**F**) AUC from three biologically independent growth curves performed as described for panel **E**. (**G**) AUC from three biologically independent growth curves performed for the indicated strains grown in Zn-replete conditions. Data are presented as mean ± SD from three biologically independent experiments. Statistical significance was determined by one-way ANOVA with Dunnett’s multiple-comparison test relative to the WT strain. ****, *P* ≤ 0.0001.

In *Y. pestis*, ZnuABC and yersiniabactin (Ybt) function redundantly to mediate Zn acquisition and are required for full virulence (47). To determine whether these systems play similar roles in *Y. pseudotuberculosis*, we analyzed mutants lacking either *irp2*, required for Ybt synthesis, or *znuA*. As previously reported for *Y. pestis* (23), inactivation of *znuA* or *irp2* alone did not significantly limit the ability of *Y. pseudotuberculosis* to grow in Zn-limited medium (Fig. 2A and B). In contrast to the *Y. pestis znuA irp2* mutant, which exhibited attenuated growth under Zn-limited conditions (23), a *Y. pseudotuberculosis znuA irp2* mutant did not exhibit growth defects under the same conditions (Fig. 2A and B). These results indicate that *Y. pseudotuberculosis* possesses (an) additional Zn acquisition mechanism(s) that compensates for the loss of ZnuABC and Ybt.

### Yersinopine is a Zn acquisition system in *Y. pseudotuberculosis* but is not required for *Y. pestis* Zn uptake

**Fig 3:**
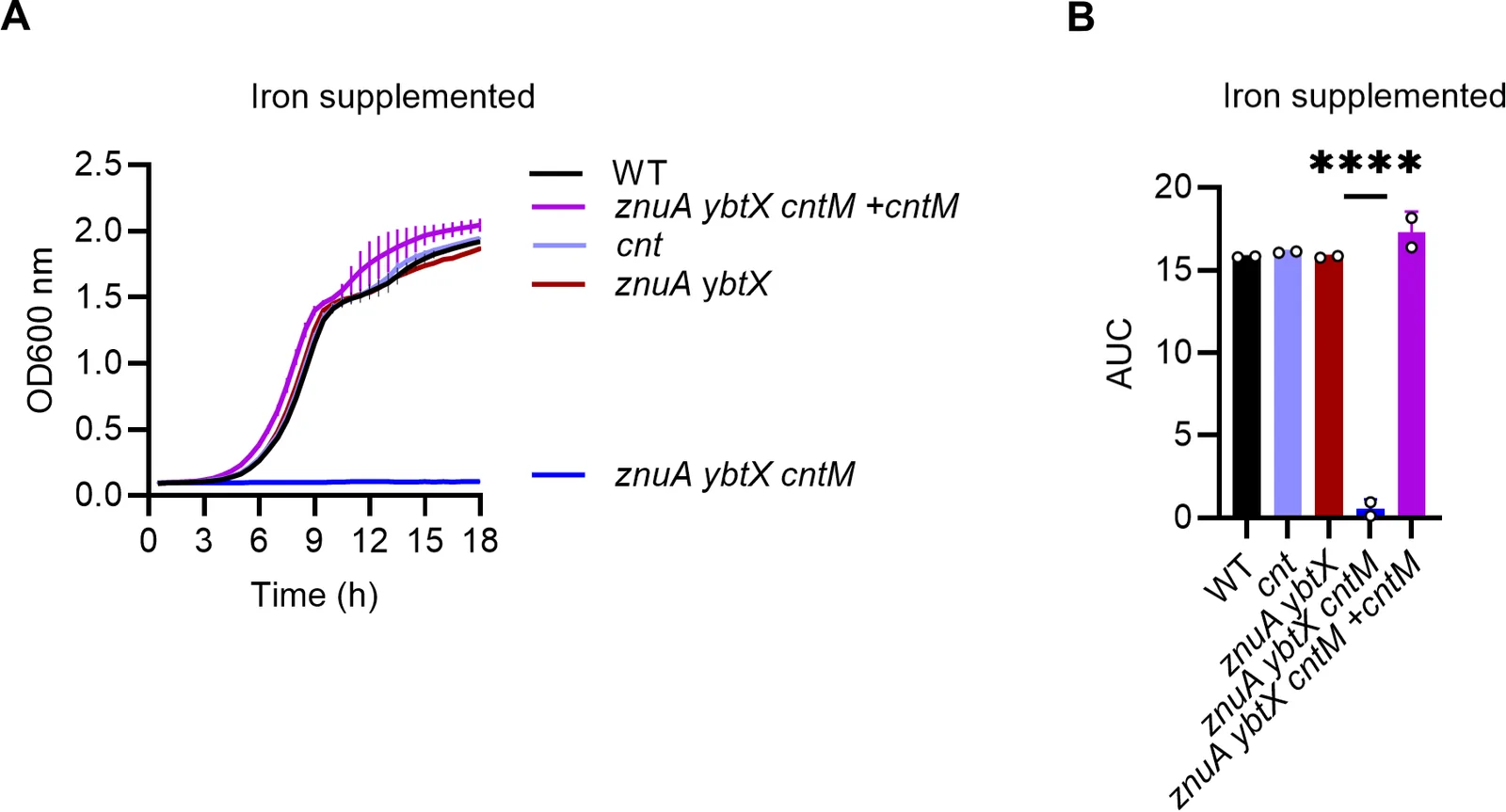
Fe supplementation unable to rescue the growth defect of *Y. pseudotuberculosis* under Zn limitation. (**A**) Representative growth curve of *Y. pseudotuberculosis* (WT) or indicated strains grown with Fe supplemented medium. (**B**) Area under the curve (AUC) from two biologically independent growth curves performed as described for panel **A**. Data are presented as mean ± SD from two biologically independent experiments. Statistical significance was determined by ordinary one-way ANOVA with Dunnett’s multiple-comparison test relative to the WT strain. ****, *P* ≤ 0.0001.

While a previous study suggested that yersinopine (Ypn), a nicotianamine-like metallophore, does not contribute to Zn acquisition in *Y. pseudotuberculosis* (32), the *cnt* operon (*cnt^PQRSLMI^*; Fig. 1), which encodes the genes for the synthesis and transport of Ypn, is highly upregulated in a *zur* mutant in both *Y. pseudotuberculosis* and *Y. pestis* (30, 31). Because Zur regulation suggests that Ypn expression is important during Zn limitation, and studies demonstrated that the closely related metallophores staphylopine and pseudopaline contribute to Zn acquisition in *S. aureus* and *P. aeruginosa*, respectively (28, 35, 48), we decided to reinvestigate if Ypn might contribute to Zn acquisition in *Y. pseudotuberculosis*. Toward this goal, we generated a clean deletion of the entire *cnt* operon and assessed growth under Zn-limited conditions. Inactivation of the Ypn biosynthetic and transport machinery alone did not significantly alter the ability of *Y. pseudotuberculosis* to grow during Zn starvation (Fig. 2A and B). However, if the *cnt* mutation was combined with either a *znuA* or *irp*2 mutation, *Y. pseudotuberculosis* growth was significantly diminished in Zn-limited medium (Fig. 2A and B; *P* ≤ 0.0001). Notably, combining all three mutations in the same background completely attenuated the ability of *Y. pseudotuberculosis* to grow under Zn limitation (Fig. 2A and B; *P* ≤ 0.0001). The growth of all three mutants was rescued by Zn supplementation (Fig. 2C and D). However, because Ybt also participates in Fe acquisition (22), and Laffont et al. suggested that Ypn might also contribute to Fe acquisition (32), we sought to exclude potential Fe acquisition-related defects from this phenotype. While *irp2* is required for both Ybt-Fe and Ybt-Zn acquisition, the inner membrane permease YbtX only mediates Ybt-Zn acquisition (i.e., *ybtX* mutants are not deficient in Fe acquisition) (23). Therefore, a *ybtX* mutation can be used to specifically determine the role of Ybt in Zn acquisition. A *znuA ybtX cnt* mutant phenocopied the severe growth defect observed in the *znuA irp2 cnt* strain under Zn limitation (Fig. 2E and F), and Zn supplementation restored growth to wild-type levels (Fig. 2G; Fig. S1). Due to difficulty in complementing the full *cnt* operon, we generated a mutant of *cntM* (required for Ypn biosynthesis) in the *znuA ybtX* background. As expected, the *znuA ybtX cntM* mutant exhibited the same phenotype as the full *cnt* deletion (Fig. 2E and F). Genetic complementation of *cntM* or Zn supplementation fully restored the growth defect of the *znuA ybtX cntM* mutant under Zn-limiting conditions (Fig. 2E and F), whereas Fe supplementation did not rescue the growth defect (Fig. 3A and B). Together, these results demonstrate that Ypn contributes to Zn acquisition in *Y. pseudotuberculosis*.

### Frameshift mutations in *cntQ* compromise yersinopine-mediated Zn uptake

**Fig 4:**
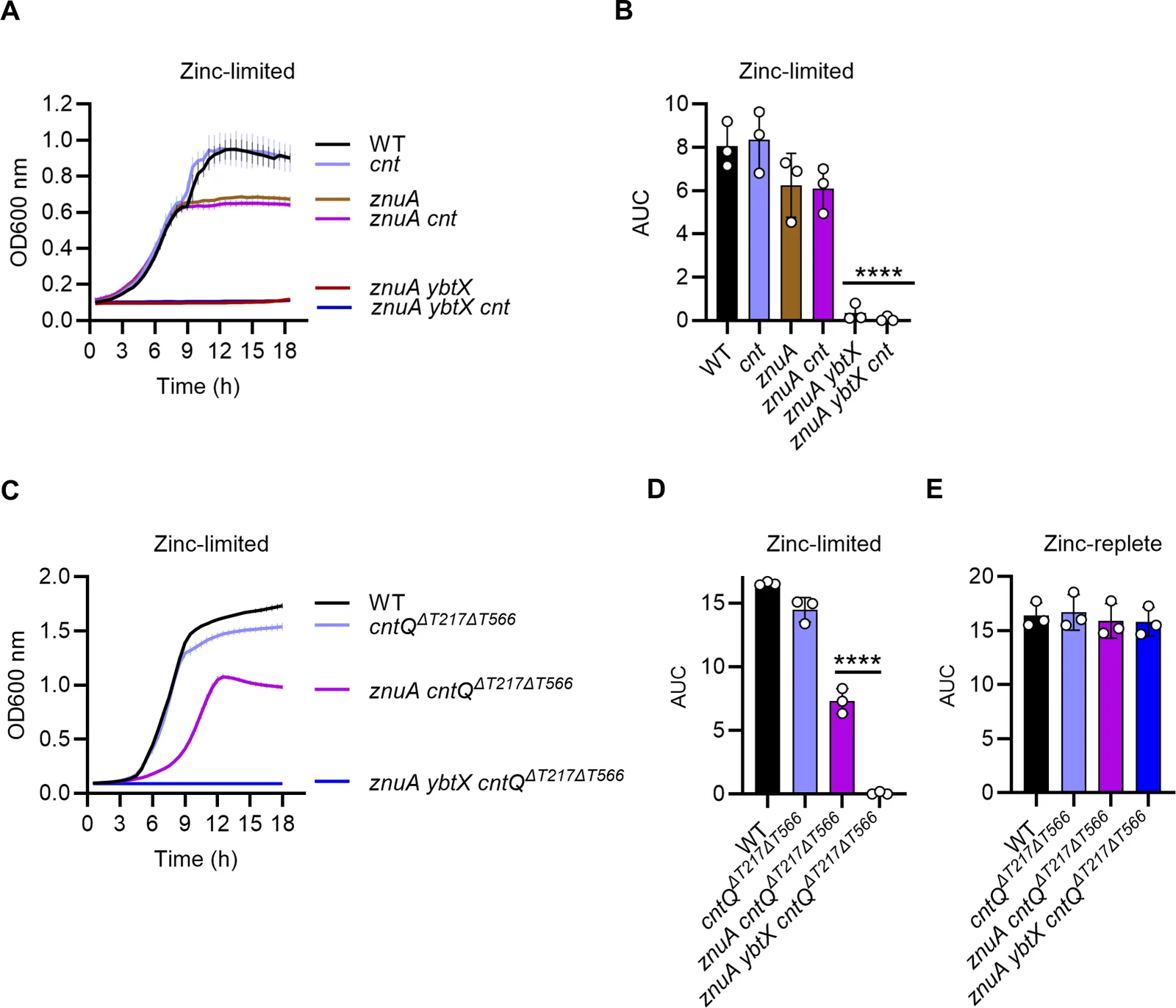
Frameshift mutations in *cntQ* compromise yersinopine-mediated Zn uptake. (**A**) Representative growth curve of *Y. pestis* (WT) and indicated mutant strains grown under Zn-limited conditions. (**B**) Area under the curve (AUC) from three biologically independent growth curves performed as described for panel **A**. (**C**) Representative growth curve of *Y. pseudotuberculosis* (WT) and *cntQ* frameshift mutants grown under Zn-limited conditions. (**D**) AUC from three biologically independent growth curves performed as described for panel **C**. (**E**) AUC from three biologically independent growth curves performed for the indicated strains grown in Zn-replete conditions. Data are presented as mean ± SD from three biologically independent experiments. Statistical significance was determined by ordinary one-way ANOVA with Dunnett’s multiple-comparison test relative to the WT strain. ****, *P* ≤ 0.0001.

The *cnt* locus is conserved in *Y. pestis* and highly upregulated in a *Y. pestis zur* mutant (30). Therefore, we next tested if Ypn contributes to Zn acquisition in *Y. pestis*. In contrast to *Y. pseudotuberculosis*, deletion of the *cnt* operon did not exacerbate the Zn-limited growth defects of the *Y. pestis znuA* or *znuA ybtX* mutants during Zn starvation (Fig. 4A and B). These results indicate that Ypn does not participate in Zn uptake in *Y. pestis*. Comparative genomic analyses revealed two frameshift mutations in the *Y. pestis cntQ* gene, which encodes the permease of an ABC transporter predicted to mediate Ypn uptake (Fig. 1) (32, 49, 50). These mutations truncate the gene product that would be produced by this gene, suggesting that *Y. pestis* is unable to import Ypn-Zn into the cell. To confirm that these mutations limit the ability to use Ypn for Zn acquisition, the same point mutations were introduced into the *cntQ* gene (*cntQ^ΔT217ΔT566^*) of *Y. pseudotuberculosis znuA* and *znuA ybtX* mutant backgrounds, and growth was evaluated under Zn-limited and replete conditions. The growth of the *cntQ^ΔT217ΔT566^* mutants phenocopied that of the *znuA ybtX cnt* and *znuA ybtX cntM* mutants (Fig. 4C and D), and Zn supplementation fully rescued these phenotypes (Fig. 4E; Fig. S2). These results demonstrate that the *cntQ^ΔT217ΔT566^* frameshift mutations in *Y. pseudotuberculosis* are sufficient to disrupt Ypn-mediated Zn acquisition, confirming that *cntQ* inactivation is responsible for the loss of Ypn contributions to Zn uptake in *Y. pestis*.

### Yersinopine contributes to *Y. pseudotuberculosis* fitness in the presence of calprotectin

**Fig 5:**
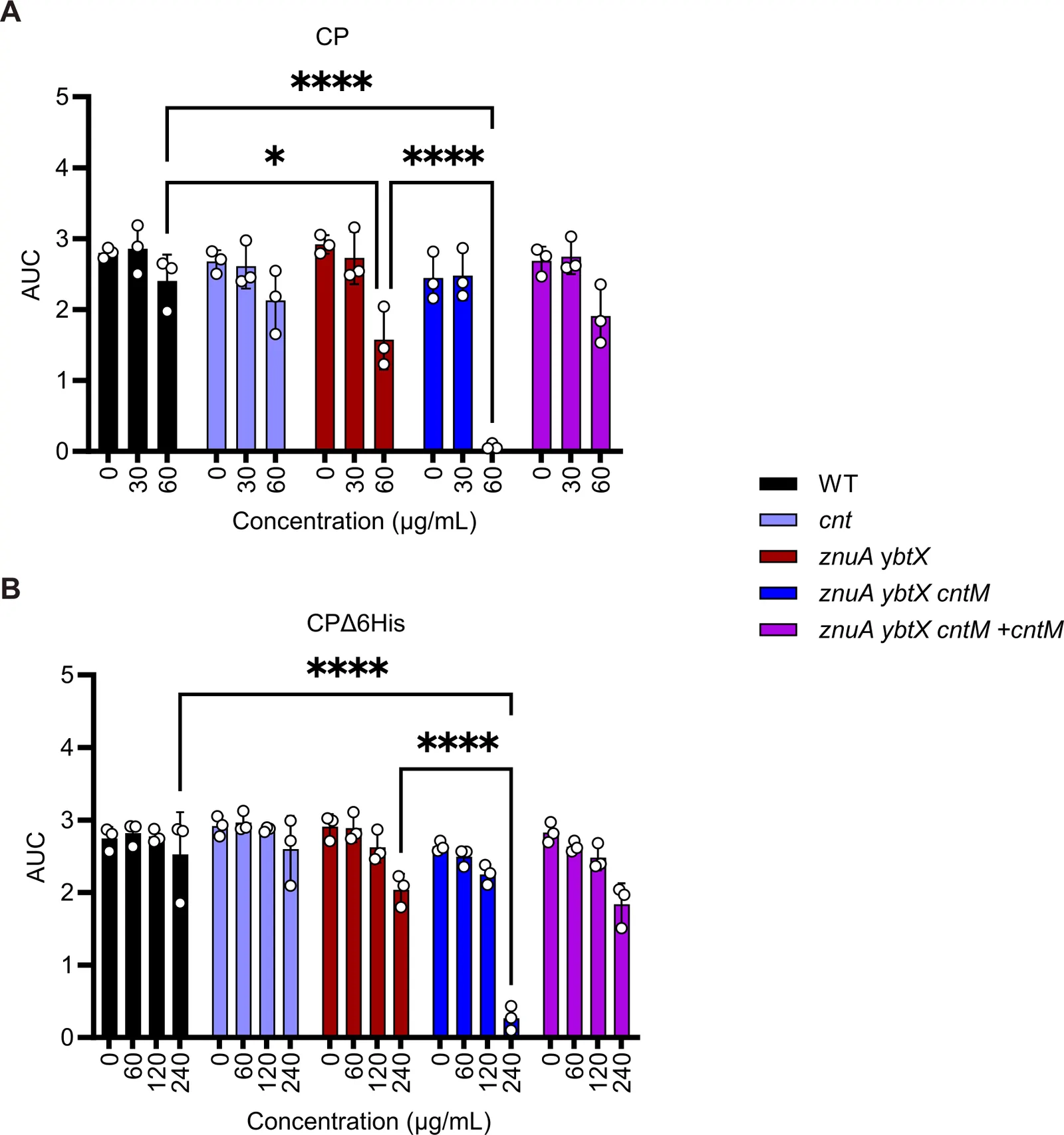
Calprotectin-mediated Zn sequestration impairs *Y. pseudotuberculosis* growth *in vitro*. Area under the curve (AUC) from three biologically independent growth curves for *Y. pseudotuberculosis* (WT) and indicated mutant strains grown in increasing concentrations of (**A**) calprotectin or (**B**) Δ6His site calprotectin. Data are presented as mean ± SD from three biologically independent experiments. Statistical significance was determined by two-way ANOVA with Tukey’s multiple-comparison test. *, *P* ≤ 0.05;****, *P* ≤ 0.0001.

Because calprotectin is secreted by host cells to sequester metals during infection, and it is a primary barrier to Zn acquisition during plague (47), we next investigated the contributions of ZnuABC, Ybt, and Ypn to *Y. pseudotuberculosis* fitness during calprotectin-mediated metal restriction. *Y. pseudotuberculosis* and mutants in each Zn acquisition system were incubated with increasing concentrations of calprotectin, and growth was measured for 18 h. As previously reported for *Y. pestis* (47), compared to the parental strain, inactivation of *znuA ybtX* resulted in attenuated growth as concentrations of calprotectin increased (Fig. 5A; Fig. S3; 60 μg/mL, *P* = 0.02). However, the *znuA ybtX cntM* mutant was more attenuated and unable to grow in the presence of 60 μg/mL calprotectin, consistent with the role of Ypn in overcoming calprotectin-mediated metal restriction (Fig. 5A; Fig. S3; *P* < 0.0001). However, because calprotectin restricts multiple metals, it was unclear if Ypn is specifically aiding in overcoming Zn restriction by calprotectin. Therefore, to determine if Ypn contributes to overcoming calprotectin-mediated Zn restriction, strains were cultured in the presence of a recombinant form of calprotectin that is unable to sequester Mn and Fe but can still sequester Zn (contains a mutation in the 6His Zn, Mn, Fe-binding site, but retains the 3HisAsp Zn-only binding site) (11, 45, 51, 52). Again, while the growth of the *znuA ybtX* mutant was modestly restricted during incubation with Δ6His site calprotectin, the *znuA ybtX cntM* mutant was more attenuated, indicating that Ypn specifically contributes to Zn acquisition in the presence of calprotectin (Fig. 5B; Fig. S4; *P* < 0.0001). For both conditions, genetic complementation with *cntM* restored growth to the same levels as the *znuA ybtX* mutant (Fig. 5A and B; Fig. S3 and S4). Finally, a *cnt*-only mutant was not significantly attenuated in the presence of calprotectin, further demonstrating that ZnuABC, Ybt, and Ypn are functionally redundant Zn acquisition systems in *Y. pseudotuberculosis*.

### Yersinopine contributes to *Y. pseudotuberculosis* virulence

**Fig 6:**
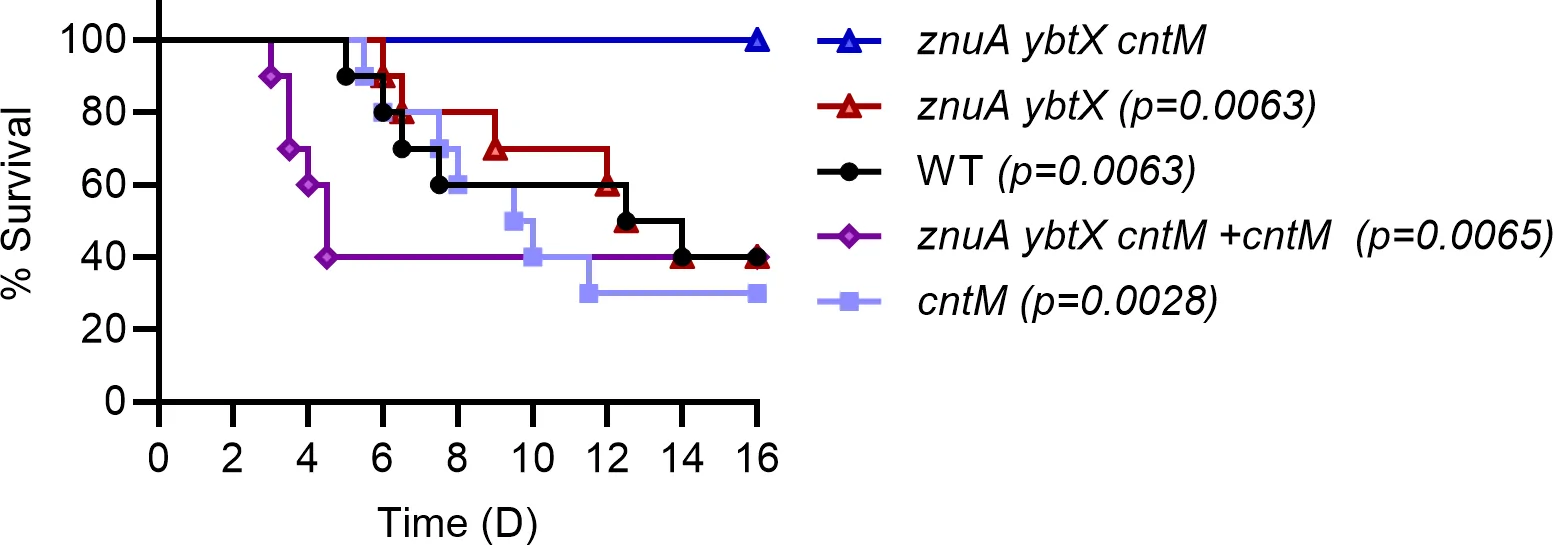
Yersinopine contributes to *Y. pseudotuberculosis* virulence. C57BL/6J mice (*n* = 10 per group) were intragastrically challenged with 10^8^ CFU of *Y. pseudotuberculosis* (WT) or indicated mutants. *znuA ybtX cntM* +*cntM* represents *cntM* genetic complementation. Statistical significance in survival distributions was determined using the log-rank test to evaluate differences in median survival. *P* values for each strain represent comparisons to the *znuA ybtX cntM* mutant strain.

Zn restriction is a potent barrier to infection (53), and *in vivo* RNAseq from *Y. pseudotuberculosis*-infected mice indicates that the *cnt* genes are significantly upregulated during infection (54). However, the contribution of Ypn during infection has not been directly investigated. To determine if Ypn contributes to fitness during yersiniosis, C57BL/6J mice were challenged intragastrically with *Y. pseudotuberculosis* or the *cntM* mutant, and the development of moribund disease was monitored for 16 days (Fig. 6). No significant differences in overall survival were observed, with both strains resulting in 60% lethality, suggesting that Ypn is not essential for virulence in this model. However, because Ypn, Ybt, and ZnuABC appear to be redundant Zn acquisition systems, we next asked if Ypn is sufficient for virulence in the absence of the other two Zn acquisition systems. Similarly to the *cntM* mutant, the *znuA ybtX* mutant was also not attenuated in C57Bl/6J mice (Fig. 6). However, the *znuA ybtX cntM* mutant was completely avirulent, with all mice surviving infection (Fig. 6; *P* = 0.0063 vs WT). Wild-type lethality was restored by genetically complementing with a functional copy of *cntM*. Together, these data demonstrate that ZnuABC, Ybt, and Ypn are functionally redundant during infection, and Ypn is sufficient for virulence in the absence of ZnuABC- and Ybt-mediated Zn acquisition.

## DISCUSSION

Transition metals are required for bacterial growth, and metal sequestration during nutritional immunity is a significant barrier to microbial infections. By using *Y. pestis* Zn acquisition mutants, we previously demonstrated that bacteria encounter calprotectin-mediated Zn sequestration in both the dermis and the lungs during bubonic and pneumonic plague, respectively (47). Similarly, metal availability is severely restricted within the inflamed gut (13, 14), and *Y. pseudotuberculosis* likely encounters Zn restriction mediated by both competition with the microbiota and host secretion of metal scavenging proteins. Therefore, like *Y. pestis*, mechanisms to acquire Zn likely contribute to *Y. pseudotuberculosis* fitness during gastrointestinal colonization and are essential for the overall virulence of the bacteria. Indeed, the *Y. pseudotuberculosis znuA ybtX cntM* mutant, which is severely deficient in Zn acquisition, is completely attenuated in the mouse model of yersiniosis.

The data presented here represent the first characterization of Ypn in the context of both Zn acquisition and virulence. While we show that three systems can be used by *Y. pseudotuberculosis* to acquire Zn, based on the growth of the double mutants, Ypn appears more efficient at Zn acquisition than the Ybt and ZnuABC systems in Zn-limited environments—significant growth defects were observed only when Ypn and one of the other systems were simultaneously disrupted, but loss of *znuA* and *ybtX*, while retaining Ypn, did not result in significant growth defects. The related nicotianamine-like metallophores pseudopaline and staphylopine also appear to be the primary Zn acquisition systems for *P. aeruginosa and S. aureus*, respectively (28, 35, 48). The high affinity of the nicotianamine-like metallophores for Zn (27) might explain why these systems are more effective at scavenging Zn than cell-associated systems like ZnuABC. Ypn, staphylopine, and pseudopaline also appear to provide a significant advantage to the bacteria when competing for Zn with host calprotectin (Fig. 5) (28, 35, 55). Calprotectin is actively secreted into the intestinal lumen during inflammation and helps to scavenge Zn during bacterial gastrointestinal infections (14). Previous work has shown that *E. coli* Nissle secretes Ybt to outcompete *Salmonella* for limited Zn resources during intestinal inflammation, demonstrating the importance of secreted metallophores in the intestinal lumen (24). Our data suggest that Ypn is sufficient to overcome calprotectin Zn sequestration *in vitro*, and because the *znuA ybtX* mutant was as lethal as wild-type *Y. pseudotuberculosis* (Fig. 5), Ypn is also sufficient to cause lethal infection in this murine model of disseminated yersiniosis, further supporting that Ypn is a primary Zn acquisition system used by *Y. pseudotuberculosis* during infection. However, maintaining multiple functionally redundant Zn acquisition systems requires an energy cost, suggesting that ZnuABC and Ybt enhance overall fitness in an environment that the laboratory models we tested here were unable to uncover. For example, functional redundancy may increase the bacteria’s ability to overcome microbiota-mediated colonization resistance, especially in the inflamed gastrointestinal environment, lowering the infectious dose or increasing shedding potential. Moreover, the systems may be expressed at different times during infection and thus contribute differently to different stages of the disease process (e.g., overcoming colonization resistance, invading the intestinal tissue, or disseminating to deeper tissues). Assessing differences in bacterial colonization of local GI tissues vs systemic tissues may be more sensitive in defining subtle fitness contributions of each system and could be used in the future to better inform if one system contributes more to a specific stage of the infection.

Recently, Laffont et al. reported data suggesting that Ypn is not involved in Zn homeostasis in *Y. pseudotuberculosis* (32). Specifically, they cultured *Y. pseudotuberculosis* and a *cnt* mutant under metal-replete conditions and observed no significant differences in intracellular Zn concentrations by inductively coupled plasma mass spectrometry. We also did not observe any growth defects of *cnt* mutants under Zn limitation if ZnuABC and YbtX were present (Fig. 2), suggesting that these two systems masked any potential Zn phenotypes in the Ypn mutant in the Laffont study (32). Studies with staphylopine also failed to initially observe Zn-associated phenotypes due to the presence of the Zn transporter AdcABC, and the importance of staphylopine in Zn homeostasis was only appreciated once the redundant system was inactivated (27, 29, 35). The authors also reported a small (~30%) but reproducible reduction in intracellular Fe levels in the *cnt* mutant and suggested that Ypn is instead an Fe acquisition system (32). However, because these studies were performed in metal-replete conditions, when the *cnt* genes are normally repressed by Zur, and in the presence of other redundant metal acquisition systems, it is difficult to clearly interpret if these data indicate that Ypn significantly contributes to Fe homeostasis. Furthermore, in our hands, Fe supplementation was unable to restore growth of the *znuA ybtX cntM* mutant (Fig. 3), while Zn supplementation completely restored growth (Fig. 2G), suggesting that a defect in Fe acquisition was not responsible for the *znuA ybtX cntM* growth phenotype. Together, these data support that Ypn is more likely a primary mediator of Zn homeostasis rather than Fe homeostasis in *Y. pseudotuberculosis*. However, while pseudopaline and staphylopine appear to also be primarily Zn transporters, they have been shown to mediate import of metals in addition to Zn (27, 29, 56). Pseudopaline binds to nickel, and pseudopaline mutants have reduced nickel accumulation (28). Similarly, staphylopine has a broad-spectrum metal-binding capacity, binding to Zn, nickel, cobalt, copper, and Fe (27), but metal binding and import are not always beneficial. Binding of staphylopine to copper can sensitize *S. aureus* 1,000-fold to copper intoxication in culture and necessitates the presence of active copper efflux systems during infection to maintain proper homeostasis (56). Therefore, while our data suggest that Ypn is primarily important for Zn homeostasis, because of the conserved structure of Ypn with these other metallophores, it is possible that Ypn also binds to other metals and could impact metal homeostasis beyond Zn. Importantly, the presence of functionally redundant acquisition mechanisms for other metals needs to be carefully considered to ensure that phenotypes are not masked in a similar fashion as we observed for Zn. Finally, while Ypn may bind multiple metals, whether these interactions are beneficial to *Y. pseudotuberculosis* also needs to be determined empirically.

*Y. pestis* recently evolved from *Y. pseudotuberculosis* to be a blood-borne pathogen that is very rarely found outside of either the mammalian or flea hosts (57). It has evolved by acquiring new virulence factors and by inactivating many genes no longer required for this new lifestyle. This evolution has resulted in a large number of pseudogenes in the *Y. pestis* genome. The two inactivating frameshift mutations in *cntQ* are conserved in all current lineages of *Y. pestis*, including the sylvatic lineages which do not cause lethal infection in humans (recently analyzed in reference 32), indicating that inactivation occurred very early during divergence from *Y. pseudotuberculosis*. This conservation suggests that the environmental conditions requiring Ypn-mediated Zn acquisition are no longer encountered by *Y. pestis*, either because they exist outside its current flea-mammalian transmission cycle or are confined to *Y. pseudotuberculosis*-specific niches such as the gastrointestinal tract or broader environment. However, because the metallophore is still produced (32) but not imported, it may still provide a yet-to-be-defined fitness advantage to *Y. pestis* independent of Zn acquisition. Alternatively, it could be that in the new environments inhabited by *Y. pestis*, Ypn uptake results in a fitness disadvantage that led to *cntQ* inactivation. Future studies with Ypn in *Y. pestis* are required to clearly understand if Ypn secretion provides a fitness advantage during plague.

*Y. pestis* still needs to overcome Zn limitation during host colonization, but it relies on ZnuABC and Ybt to compete for Zn with calprotectin in the mammalian host and acquire Zn during colonization in the flea midgut (47). However, because it is unable to use Ypn to acquire Zn, *Y. pestis* is more sensitive to Zn limitation than *Y. pseudotuberculosis*, which is highlighted by attenuated growth of the *Y. pestis znuA* mutant in Zn-limited medium relative to *Y. pseudotuberculosis* (Fig. 2 and 4). *Y. pestis znuA* growth in Zn-limited medium more closely phenocopies the growth of *Y. pseudotuberculosis znuA cnt*. The ability of *Y. pestis* to rely on only ZnuABC or Ybt to overcome calprotectin-mediated sequestration during plague further suggests that Zn acquisition redundancy, and specifically Ypn-mediated Zn acquisition, may be more crucial for *Y. pseudotuberculosis* colonization of the gastrointestinal tract rather than systemic disease.

Collectively, these data have significantly improved our understanding of Zn homeostasis in *Y. pseudotuberculosis* and revealed that Ypn is primarily a Zn acquisition system. Moreover, while Ypn is functionally redundant with two other Zn acquisition systems, it is sufficient on its own to mediate Zn homeostasis and virulence in the mammalian host. Finally, this study has revealed a fundamental divergence in Zn acquisition strategies between *Y. pseudotuberculosis* and *Y. pestis*, reflecting evolutionary adaptation to distinct ecological niches and host environments.

## SUPPLEMENTAL MATERIAL

Supplemental material mbio.02000-26-s0001.pdf Tables S1−S3; Fig. S1−S4.

ASM does not own the copyrights to Supplemental Material that may be linked to, or accessed through, an article. The authors have granted ASM a non-exclusive, world-wide license to publish the Supplemental Material files. Please contact the corresponding author directly for reuse.
