# Supplementary material for: Yersinopine is a primary zinc acquisition system for Yersinia pseudotuberculosis
Source: mBio. 2026 Sep 17;17(10):e02000-26. doi: 10.1128/mbio.02000-26 (PMC13712977; doi:10.1128/mbio.02000-26)
Supplement: Supplementary file 1 [file mbio.02000-26-s0001.pdf]

Supporting information

Table S1. *Y. pseudotuberculosis* strains (pYV^+) used in this study			
**Descriptive Name**	**Genotype**	**Identification Number**	**Source**
Y. pseudotuberculosis IP32953	Pgm^+ pYV^+	MBL 256	Gift from Dr. Ralph Isberg [1]
cnt	MBL 256 *Δcnt*^{PQRSLMI}	LOUY029	In this study
znuA	MBL 256 *ΔznuA*	LOUY039	In this study
cnt znuA	LOUY029 *ΔznuA*	LOUY040	In this study
ybtX	MBL 256 *ΔybtX*	LOUY043	In this study
znuA ybtX	LOUY039 *ΔybtX*	LOUY044	In this study
cnt irp2	LOUY029 *Δirp2*	LOUY045	In this study
znuA irp2	LOUY039 *Δirp2*	LOUY046	In this study
irp2	MBL 256 *Δirp2*	LOUY047	In this study
cntQ	MBL 256 *ΔcntQ*	LOUY053	In this study
cnt irp2 znuA	LOUY045 *Δirp2*	LOUY055	In this study
cnt znuA ybtX	LOUY040 *ΔybtX*	LOUY056	In this study
cntQ^{ΔT217ΔT566}	LOUY053 *cntQ*^{ΔT217ΔT566}	LOUY058	In this study
cntQ^{ΔT217ΔT566}*znuA*	LOUY058 *ΔznuA*	LOUY069	In this study
cntQ^{ΔT217ΔT566}*znuA ybtX*	LOUY069 *ΔybtX*	LOUY072	In this study
cntM	MBL 256 *ΔcntM*	LOUY075	In this study
znuA ybtX cntM	LOUY044 *ΔcntM*	LOUY76	In this study
znuA ybtX cntM +cntM	LOUY76 *c:cntM*	LOUY77	In this study
***Y. pseudotuberculosis* strains (pYV^-) used in this study**			
Y. pseudotuberculosis IP32953	MBL 256 pYV^-	LOUY016	In this study
cnt	LOUY029 pYV^-	LOUY030	In this study
znuA	LOUY039 pYV^-	LOUY048	In this study
cnt znuA	LOUY040 pYV^-	LOUY052	In this study
znuA ybtX	LOUY044 pYV^-	LOUY060	In this study
cnt irp2	LOUY045 pYV^-	LOUY051	In this study
znuA irp2	LOUY046 pYV^-	LOUY050	In this study
irp2	LOUY047 pYV^-	LOUY049	In this study
cntQ	LOUY053 pYV^-		In this study
cnt irp2 znuA	LOUY055 pYV^-	LOUY057	In this study
cnt znuA ybtX	LOUY056 pYV^-	LOUY061	In this study
cntQ^{ΔT217ΔT566}	LOUY058 pYV^-	LOUY070	In this study
cntQ^{ΔT217ΔT566}*znuA*	LOUY069 pYV^-	LOUY071	In this study
cntQ^{ΔT217ΔT566} *znuA ybtX*	LOUY072 pYV^-	LOUY073	In this study
znuA ybtX cntM	LOUY76 pYV^-	LOUY078	In this study
znuA ybtX cntM +cntM	LOUY77 pYV^-	LOUY079	In this study

**Table S2. *Y. pestis* strains used in this study**			
**Descriptive Name**	**Genotype**	**Identification Number**	**Source**
Y. pestis KIM6+	pCD1$^-$, Pgm$^+$, pMT$^+$, pPCP1$^+$	YPA 152	[2]
znuA	YPA 152 *ΔznuA*	YPA175	[3]
znuA ybtX	YPA175*ΔybtX*	YPA177	[3]
cnt	YPA 152 *Δcnt*^{QPQRSLMI}	YPA198	In this study
cnt znuA	YPA198 *ΔznuA*	YPA374	In this study
cnt znuA ybtX	YPA374 *ΔznuA*	YPA378	In this study
***E. coli* strains used in this study**			
E. coli S17 with 1λPIR. This will maintain plasmids with an R6k origin of replication			

**Table S3. Primers used in this study**		
**Descriptive Name**	**Primer Sequence (5' → 3')**	**Identification Number**
cnt^{PQRSLMI} KO 5' F	GCA GCC CGG GGG ATC GGA ACG GAG AGT TAC	DNA796
cnt^{PQRSLMI} KO 5' R	GCT CTA GAA CTA GTG CCA CTC AAC TGT TCT	DNA797
cnt^{PQRSLMI} KO 3' F	TCC TTT ATG TTA TTA TGA TAT TCA CAC AAG	DNA798
cnt^{PQRSLMI} KO 3' R	CTT GTG TGA ATA TCA TAA TAA CAT AAA GGA	DNA799
znuA KO 5' F	TCG ATA AGC TTG ATA TCG AAT TCC T GTG TCA TTC ACT GTC CAG GAG	DNA1047
znuA KO 5' R	GGC TAA AAC AGG CAA TGC TC TCT CAG TTG TCG AAC CAA TAT GTG	DNA1048
znuA KO 3' F	CAC ATA TTG GTT CGA CAA CTG AGA GAG CAT TGC CTG TTT TAG CC	DNA1049
znuA KO 3' R	CGA AAT TAA CCC TCA CTA AAG GGA AC ATC CAA CAC CAG CAA CTG TG	DNA1050
ybtX KO 5' F	TCG ATA AGC TTG ATA TCG AAT TCC TTT GCT CAG GGG ATG ATG GTA TTG C	DNA1188
ybtX KO 5' R	GCT TGT CAT CAG TTC TCT TGC CGA CGA TAC CAG ACT CTG CGT CAC ATA	DNA1189
ybtX KO 3' F	TAT GTG ACG CAG AGT CTG GTA TCG TCG GCA AGA GAA CTG ATG ACA	DNA1190
ybtX KO 3' R	CGA AAT TAA CCC TCA CTA AAG GGA ACA AAC AGC ACG GTA AAC GCA TCC	DNA1191
irp2 KO 5' F	TCGATAAGCTTGATATCGAATTCCTCATTGCCC ATCAGAGATTGCAGACC	DNA1250
irp2 KO 5' R	GTAAGACGTGCCATCAGGAGGAAGACGCGAA GCAAACTGATTTTCCCCGG	DNA1251
irp2 KO 3' F	CCGGGGAAAATCAGTTTGCTTCGCGTCTTCC TCCTGATGGCACGTCTTAC	DNA1252
irp2 KO 3' R	CGAAATTAACCCTCACTAAAGGGAACACGCTA TGGTTGCAGGGGCAAAGT	DNA1253

cntQ KO 5' F	TCGATAAGCTTGATATCGAATTCCTCCATCGC CTATCAACAAGGCATTGC	DNA1281
cntQ KO 5' R	CGTGCTTTAACGTCAGTTGCGTCATAGCGGC CAGTAGCGAGCCGATCAAT	DNA1282
cntQ KO 3' F	ATTGATCGGCTCGCTACTGGCCGCTATGACG CAACTGACGTTAAAGCACG	DNA1283
cntQ KO 3' R	CGAAATTAACCCTCACTAAAGGGAACCATGAC AGGCAACATTTCCACGGT	DNA1284
cntQ$^{\Delta T217}$ F	CACCAGATCATTTTTGGTCCATTCGTTTGCCG ATGACGTTAACCGCCGTG	DNA1312
cntQ$^{\Delta 217}$ R	GCAAACGAATGGACCAAAAATGATCTGGTGTA GTTCATCATGCTGCGAAG	DNA1313
cntQ$^{\Delta T566}$ F	CACTCATGTTATTTCGGCCTCACCAGAAGCGA TGCAACAAATTGTATTCTG	DNA1314
cntQ$^{\Delta T566}$ R	GCTTCTGGTGAGGCCGAAATAACATGAGTGA TTGCAACGACTGGAAGAAG	DNA1315
cntM KO 5' F	TCGATAAGCTTGATATCGAATTCCTTCCAAGA CTGACGACTACCTGACAAG	DNA1337
cntM KO 5' R	TAGCCAATAACGCGCCTTGGTTCATGTGTTAT GCATGATGATTAGGTTC	DNA1338
cntM KO 3' F	GGAACCTAATCATCATGCATAACACATGAACC AAGGCGCGTTATTGGCTA	DNA1339
cntM KO 3' R	CGAAATTAACCCTCACTAAAGGGAACTCAGG CCGCCTTCTTTTTACTGCG	DNA 1340

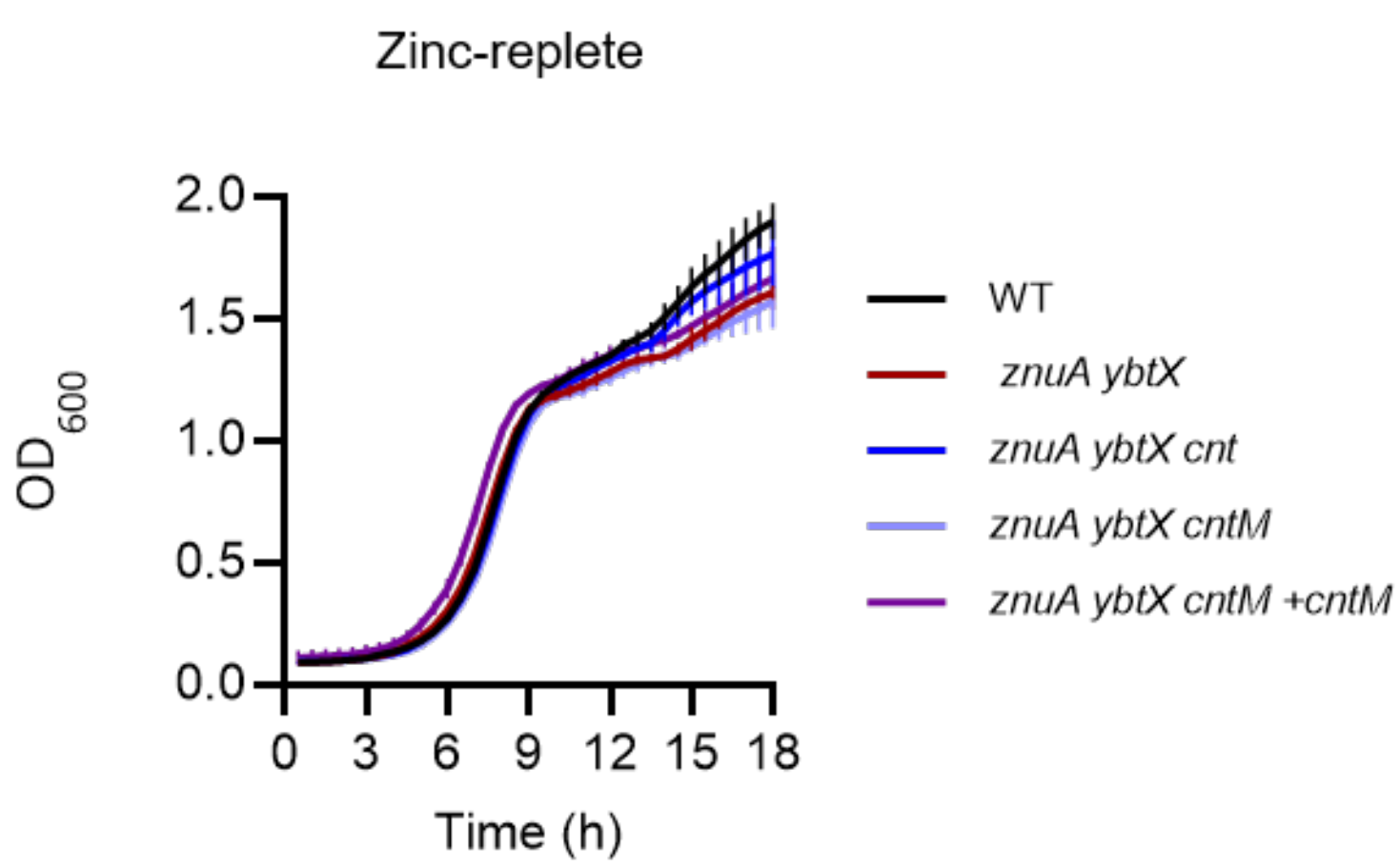

Figure S1. Representative growth curve of *Y. pseudotuberculosis* (WT) and indicated strains grown under Zn-replete conditions.

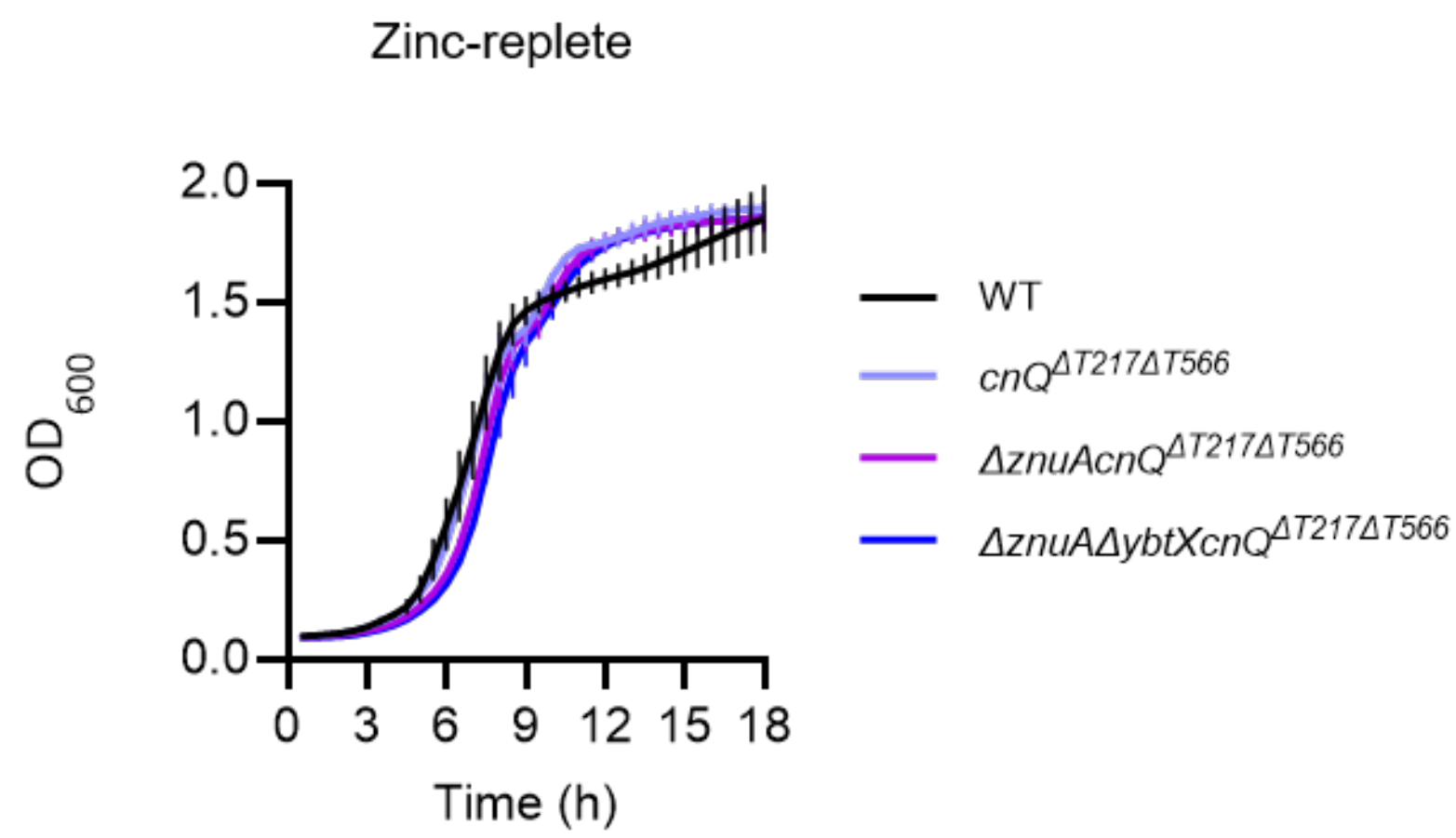

Figure S2: Representative growth curve of *Y. pseudotuberculosis* (WT) and *cntQ* frameshift mutants grown under Zn-replete conditions.

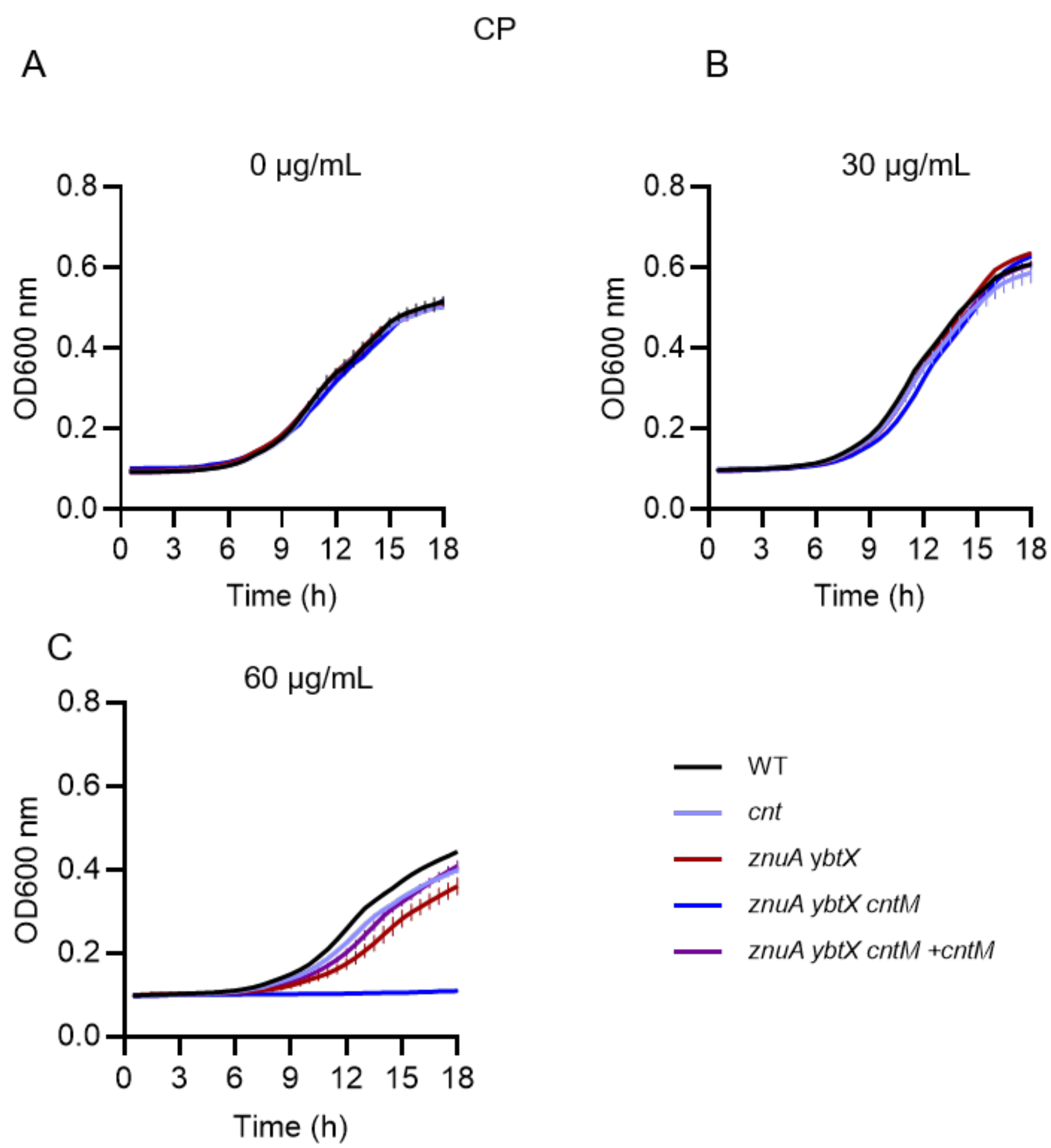

Figure S3: Representative growth curve of *Y. pseudotuberculosis* (WT) and indicated strains grown in (A) the absence of calprotectin, or with (B) 30 µg/ml, or (C) 60 µg/ml calprotectin.

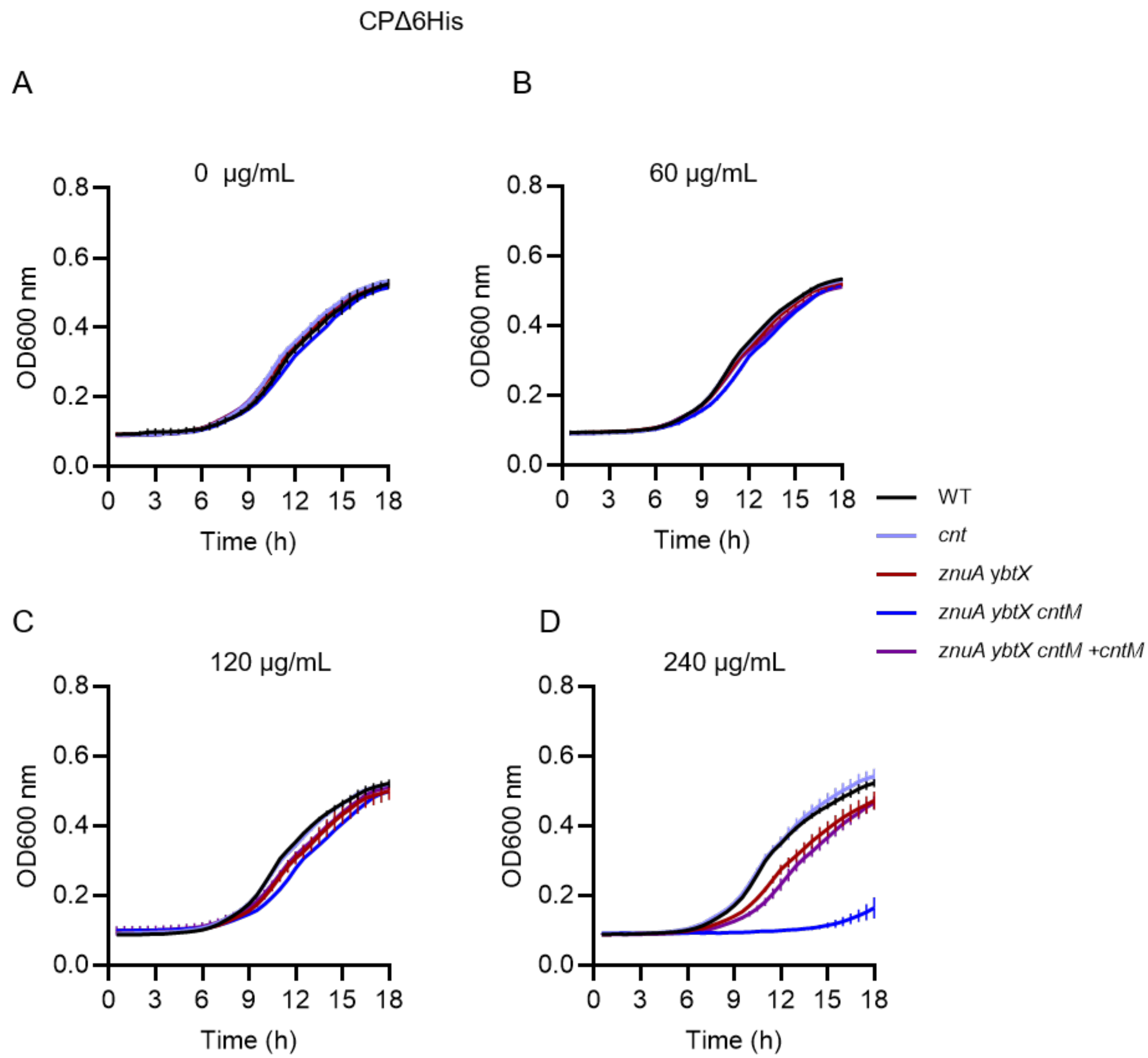

Figure S4: Representative growth curve of *Y. pseudotuberculosis* (WT) and indicated strains grown in (A) the absence of Δ6His calprotectin, or with (B) 60 μg/ml, (C) 120 μg/ml, or (D) 240 μg/ml Δ6His calprotectin.

References

1. Chain, P.S., et al., *Insights into the evolution of Yersinia pestis through whole-genome comparison with Yersinia pseudotuberculosis.* Proc Natl Acad Sci U S A, 2004. **101**(38): p. 13826-31.
2. Fetherston, J.D., P. Schuetze, and R.D. Perry, *Loss of the pigmentation phenotype in Yersinia pestis is due to the spontaneous deletion of 102 kb of chromosomal DNA which is flanked by a repetitive element.* Mol Microbiol, 1992. **6**(18): p. 2693-704.
3. Bobrov, A.G., et al., *Zinc transporters YbtX and ZnuABC are required for the virulence of Yersinia pestis in bubonic and pneumonic plague in mice.* Metallomics, 2017. **9**(6): p. 757-772.
